# Identification of elite performance characteristics in a small sample of taekwondo athletes

**DOI:** 10.1371/journal.pone.0217358

**Published:** 2019-05-31

**Authors:** Mohd Rozilee Wazir Norjali Wazir, Maxim Van Hiel, Mireille Mostaert, Frederik J. A. Deconinck, Johan Pion, Matthieu Lenoir

**Affiliations:** 1 Faculty of Medicine and Health Sciences, Department of Movement and Sports Sciences, Gent, Belgium; 2 Department of Sport Studies, Faculty of Educational Studies, University Putra Malaysia, Serdang, Selangor, Malaysia; 3 HAN University of Applied Sciences, Nijmegen, Netherlands; James Cook University College of Healthcare Sciences, BRAZIL

## Abstract

Along with the increasing popularity of taekwondo, there is a need of evidence-based talent identification (TID) and development programs based upon profiles of future elite athletes. This study first aims to investigate the differences between elite and non-elite taekwondo athletes in anthropometry, physical performance and motor coordination. The second aim is to demonstrate the applicability of z-scores in TID research. A total of 98 Taekwondo athletes between 12 and 17 years old were tested using a generic test battery consisting of four anthropometrical (Height, Weight, Fat Percentage, BMI), six physical performance (Sit & Reach, Sprint 5m, Sprint 30m, Counter Movement Jump, Squat Jump, Endurance Shuttle Run) and three motor coordination tests (Moving Sideways, Jumping Sideways, Walking Backwards). Based on the individual success at international competition level, 18 were categorised as elite athletes and 80 were considered as non-elite. T-tests (step 1) on raw test scores and MANOVAs on z-scores (step 2) were conducted to examine differences between the elite and non-elite taekwondo athletes for anthropometry, physical performance and motor coordination tests. Finally, z-scores were reconverted to raw scores to demonstrate practical significance for coaches. Overall, elite taekwondo athletes score better compared to the non-elite group. The MANOVA analysis better scores for elites on fat percentage (-0.55 versus 0.12;p = 0.006), BMI (-0.37 versus 0,08;p = 0.067) sprint speed 30m (-0.48 versus 0.11;p = 0.029), counter movement jump (0.79 versus -0.18;p = 0.000), squat jump (0.42 versus -0.11;p = 0.041), moving sideways (0.79 versus -0.18;p = 0.000) and walking backwards (0.54 versus -0.12;p = 0.006). This study confirms our knowledge on physical profiles of elite taekwondo athletes and expands our knowledge to the domain of motor coordination. This study showed how the z-score method can be used to distinguish between elite and non-elite athletes, the former being low in number by definition.

## Introduction

Combat sports like fencing, judo, taekwondo, and many others have become more popular in the past decades, with increasing number of participants around the world [1]. Along with this increased popularity, coaches and federations are in need of evidence-based talent identification and development programs. The key question in talent identification is to decide which athlete has the most potential to perform well and be successful at the highest competitive level [2]. Resources for the development of young talented athletes are limited and yet most federations are expected to provide a return on investment in the talent identification process [3]. Extensive knowledge on characteristics of combat athletes of different ages and performance levels are therefore crucial. In the past decades, profiles of athletes active at various levels of participation and in different disciplines of combat sports like wrestling [4], judo [5,6], taekwondo [6,7] and fencing [8] have been partially documented.

Taekwondo has been a part of the Olympic demonstration program since Seoul 1988 and Barcelona 1992, before becoming an Olympic discipline from the Sydney 2000 Olympic Games. In this paper we focus on the anthropometric, physical, and coordinative profiles of taekwondo athletes of different levels. Taekwondo is a full contact free-sparring sport, which consists of punches and kicks that have to produce a displacement of the body segment of the opponent. The words ‘Taekwondo’ translates as tae to hit using the foot (kick), kwon to hit using the fist (punch) and do referring to the art [9]. It is a native Korean fighting art that originated thousands of years ago and has become a popular sport with over 120 million children and adults actively participating worldwide [1]. Taekwondo consists of 3 x 2-minute rounds with a 1-minute rest period between each round. Points are awarded for body and head contacts, matches are won via knockout or via higher point score.

The identification and selection of elite athletes is a complex process affected by several factors that vary in relation to the specific nature of the sport discipline. With respect to body height, lengths between 170 cm [10] and 183 cm [7] for adult male elite athletes are reported, however accompanied by relatively high standard deviations. Body weights range significantly between 60 and 75 kg in males and between 47 and 59 kg in female adult athletes. This large variability is at least partially related to the weight categories in taekwondo, but also within a weight category athlete of different lengths can excel. With respect to BMI and fat percentage, BMI around 21 kg/m^2^ and fat percentage around 10% for males appear to be associated with elite performance [11].

With respect to physical performance, taekwondo requires athletes to dispose of explosive leg power, and flexibility because of the emphasis on kicking [12,13,14,15,16]. Taekwondo is renowned for its swift kicks and dynamic footwork. One of the most popular used skills is roundhouse kick [15] also known as Dollyo Chagi [17] in Korean language, and other common techniques include side kick (Yop Chagi), back kick (Momdollyo Chagi) and spinning kick (Dolmyo Chagi) [16,17]. In taekwondo, specific indications of leg power derive from assessments of jumping and sprinting activities that are necessary to generate powerful kicks [12,13] and counter-movement jump (CMJ) performances have been used to evaluate leg power [18]. Leg power seems to be an important factor for proper execution of techniques while kicking or performs footwork during competition and might an indicator to distinguish between the elite and sub-elite. Lower limb explosivity is usually evaluated by means of the CMJ, in which values close to or above 40 cm are attained in males [11]. Taekwondo athletes with good lower limb explosivity will produce more power during kicking and this will give them advantages during competition.

Taekwondo belongs to a group of sports in which speed of execution plays a key role in achieving success [7,19]. The speed of taekwondo has been examined using field-test methods, including 20m sprint [15], 30m sprint [20] and 6s sprint tests [21]. Limited data from previous studies demonstrate that successful male taekwondo juniors record 4.62s (medallists) and 4.81s (non-medallist) in 30m sprint test [20]. Similarly, female medallists can run 20m in 3.6s on average, versus 3.81s for non-medallists [15]. Altogether, with respect to physical characteristics it appears that the combination of excellent speed and power values will be a great advantage for taekwondo athletes and enable the discrimination of taekwondo athletes of different competition levels.

While the morphological and physical profile of taekwondo athletes are well documented, motor coordination is virtually absent in this literature. Nevertheless, the technical requirements of taekwondo skills are high, and these skills need to be executed under severe time pressure. While the qualitative evaluation of skill execution is sometimes used in talent identification, those results are strongly affected by training history, and thus do not allow a clear evaluation of an athlete’s potential to excel in the future. Recent studies have shown that general, non-sport specific motor coordination tests can distinguish between athletes in different sports and levels of participation, and even allow the prediction of future performance [21]. The underlying idea is that an excellent coordination facilitates the acquisition of new and complex techniques. Differences in motor coordination between athletes of different levels have been found in previous studies. Pion et al. (2015) [22], demonstrated that a general motor coordination test was a stronger predictor of future performance in female elite volleyball players compared to physical test scores or specific volleyball skills like jumping and overhead throwing actions. This also indicates that non-sport specific sport test (motor coordination) should also be taken into account in detecting talented young athletes. In spite of the potential significance of coordination tests, virtually none of the studies available reports on this aspect so far in combat sports.

Information on anthropometric, physical performance and motor coordination profiles of elite level athletes is beneficial to coaches or teachers as it can be used as a reference to planning athlete training programmes and distinguishing their athletes in accordance to their data. Although these characteristics are not the only determinants of success, they do serve to provide additional input for coaches and may aid in optimizing their athlete’s potential. While literature already provides a clear picture of anthropometric and physical profiles of elite athletes, most of them are dominantly descriptive in nature. Only a few studies have focused on the comparison between elite and sub-elite athletes, and most of them do not take into account motor coordination. Therefore, this study aims to investigate the differences between elite and non-elite taekwondo athletes in anthropometry, physical performance and motor coordination. Building upon the international success of the Belgian Taekwondo athletes, the aim of this study is to identify the generic, that is, non-sport specific characteristics associated with high performance level.

In addition, this study aims at demonstrating the applicability of z-scores in TID research. The number of elite youth athletes is limited by definition, and profiles might be confounded by differences in age and gender. Conversion into z-scores based upon on external reference population might be at least partially resolve this issue.

## Material and methods

### Participants and design

The data for this study is part of the Flemish Sport Compass (FSC), a project that began in 2007 and is still on-going [23,24,25,26]. Out of a sample of 98 Taekwondo athletes age measured between 12 to 17-year-old, 18 were categorised as elite athletes based on their performance level as senior. Elites were a member of the Belgian national team (representing Belgium at international championships or at the Olympic games) and/or had won at least one medal at an international taekwondo competition. A total of 80 taekwondo athletes were considered as non-elite since they were not in the elite squad and did not participate or did not win medals in an international championship (Table 1). This study has been conducted in accordance with recognized ethical standards [27] and was approved by the local Ethics Committee of the Ghent University Hospital [28]. For all participants, written informed parental consent was obtained. None of the participants refused participation.

**Table 1:** Demographic information of the elite and non-elite taekwondo athletes.

Age Group (years)	Elite		Non Elite		Total
	Male	Female	Male	Female	
12	1	1	8	2	12
13	1	1	7	6	15
14	3	2	10	10	25
15	2	2	7	12	23
16	1	2	10	3	16
17	1	1	1	4	7
Total	9	9	43	37	98

### Measurements

The athletes were measured between 2008–2016 and completed four anthropometrical, six physical performance and three motor coordination tests. A team of experienced examiners from the Department of Movement and Sports Sciences assessed the generic, i.e. not containing sport specific tests, battery. At any given time, instruction and demonstration were standardized according to the test guidelines [29]. The athletes performed all tests barefoot with the exception of the sprints, the counter movement jump, and the endurance shuttle run test, which were all performed with running shoes.

### Anthropometry

Body height was measured using a calibrated stadiometer (0.1 cm, Harpenden, portable Stadiometer, Holtain, UK). In addition, body weight and body fat percentage were assessed using a digital balance scale with a foot-to-foot bioelectrical impendence system (0.1 kg, Tanita, BC-420SMA) according to previously described procedures [27] and manufacturer guidelines. Body height and body weight values were used to calculate Body Mass Index (BMI in kg/m^2^). In addition, sitting height was measured to calculate the Age of Peak Height Velocity (APHV) as a measure of biological maturation [30,31].

### Physical performance

Flexibility was assessed by the sit-and-reach test of the Eurofit test battery with an accuracy of 0.5 cm [25]. Speed was evaluated by two maximal sprints of 30 meters with split time measured at 5 meters. The recovery time between each sprint was set at two minutes. The fastest time for the 5m sprint and 30m sprint was used for analysis [32]. The counter movement jump (CMJ) and the squat jump were performed to estimate explosive leg power. The athletes performed three single jumps without arm swing recorded with an OptoJump device (MicroGate, Italy) and the highest of three jumps was used for further analysis (0.1 cm). Finally, the cardiorespiratory endurance was measured using the endurance shuttle run test (Beep test) with an accuracy of 0.5 min [25].

### Motor coordination

Gross motor coordination was evaluated by the short form of the “KörperkoordinationsTest für Kinder” (KTK) [29]. First, participants had to walk backwards along balance beams of decreasing width (6 cm; 4.5 cm and 3 cm respectively). Secondly, participants had to perform two-legged jumps sideways over a wooden slat (2 x 15 s), summing the number of jumps over the two trials. Thirdly, participants had to move sideways on wooden platforms (2 x 20 s), summing the number of relocations over two trials. This test has been shown to allow discrimination between combat athletes of different disciplines in previous studies [6].

### Statistical analyses

Data was analysed using SPPS for Windows version 25.0. The present study had a cross-sectional design, involving two study groups: elite and non-elite. The basic descriptive indicators (mean and standard deviation) were calculated for all analysed variables given that most of the variables were normally distributed and thus met the conditions of parametric analysis. Given the relatively low number of athletes in the elite group, which is an inherent characteristic of research in elite athletes, a three-step analysis was used. First, a t-test was applied to compare anthropometry, physical performance, and motor coordination between elites and sub-elites. Mean scores and statistical results are presented in Table 2.

**Table 2:** Data for the counter movement jump for boys in The Elite Sport Schools [23].

Age (years)	N	Minimum (cm)	Maximum (cm)	Mean (cm)	SD (cm)
12	48	18.80	38.85	27.53	4.30
13	60	17.60	38.00	27.78	4.21
14	74	19.42	51.63	31.68	5.30
15	74	23.2	54.70	34.84	5.44
16	82	24.38	51.36	35.79	5.87
17	87	25.8	56.51	37.81	6.55

The data based on the CMJ score from the Flemish Top Sport School athlete in gymnastic, skating, soccer, athletics, badminton, basketball, handball, judo, fencing, taekwondo, table tennis, triathlon, tennis, golf, volleyball, swimming, cycling, ski.

Overall, gender and age were equally distributed over both groups (see Table 1), although not for each age group separately, the analysis above is limited in that it does not take into account these factors. To allow the comparison of the results of taekwondo athletes from different age and gender groups, standardized z-scores (i.e. the difference between an individual score from the mean, divided by the standard deviation of the sample) [33] were calculated for each of the 13 variables in the second step. To this end a large reference database of 699 young, well-trained athletes in 18 sport disciplines from the Flemish Top Sport Schools of which the same measurements were available was used [23]. For example, the score on the sprint of a 12-year old male taekwondo athlete was converted into a z-score based upon all 12-year old male athletes in the Flemish Top Sport Schools, resulting in a gender- and age-neutral score. In this way, the statistical issue of low sample sizes that is inherently related to research in individual elite athletes was avoided.

Z-scores are however an abstract concept that is often not very informative neither of great practical applicability for coaches. Therefore in a third step, the obtained z-scores were reverse calculated in order to obtain the average value a taekwondo elite athlete (male or female) between 12 and 17 years should obtain. For example, Table 2 shows the mean and average scores of the counter movement jump in boys between 12 and 17 years of age from the reference population. A z-score of +1 is then equal to 27.53 + 4.30 = 31.83 cm for a 12-year old well-trained male individual. In addition, ROC analyses were performed to evaluate the discriminative power of each test separately. A high area under the curve (AUC) reflects a better discrimination of elites versus sub-elites with a given test [34].

The magnitude of the differences between the levels was estimated using Partial Eta Squared with cut-off of 0.01 (small), 0.06 (moderate) and 0.14 (large) [23]. The level of significance was set at p<0.05.

## Results

### Anthropometry

Table 3 shows the raw scores and the t-test results of the anthropometric measurements height, weight, fat percentage and body mass index in elite and non-elite athletes. The absolute values indicate that elite athletes tend to be taller and leaner compared to non-elites. The t-test results also indicate a significant difference between elite and non-elite in fat percentage as shown in Table 3. However, statistical analysis of the z-scores led to lower values in fat percentage and boarder-line lower BMI values only (Table 4). AUC for fat percentage and BMI were 0.71 and 0.74, respectively, indicating high discriminative power.

**Table 3:** Mean and standard deviations (Sd) from the descriptive analysis, T-test results and Levene’s test for elite and non-elite taekwondo athletes.

Measurement	Mean (SD)		t-Test result		Levene’s Test
	Elite	Non-Elite	t	p	p
Anthropometry					
Height (cm)	166.7 (8.28)	163.4 (10.49)	1.28	0.20	0.18
Weight (kg)	51.3 (9.00)	51.9 (10.64)	-0.19	0.85	0.42
Fat Percentage (%) *	11.9 (3.65)	15.12 (6.55)	-2.01*	0.05	0.00
BMI (kg/m^2^)	18.3 (1.78)	19.3 (2.38)	-1.59	0.12	1.22
Physical Performance					
Sit & Reach (cm)	32.5 (6.29)	30.1 (7.87)	1.22	0.23	4.22
Sprint 5m (s)	1.16 (0.08)	1.19 (0.09)	-1.59	0.12	0.72
Sprint 30m (s) *	4.72 (0.33)	4.92 (0.37)	-2.11*	0.04	0.66
Counter Movement Jump (cm) **	33.4 (4.95)	28.4 (5.62)	3.50**	0.00	0.84
Squat Jump (cm) *	30.1 (6.45)	26.4 (5.19)	2.49*	0.02	0.47
Endurance Shuttle Run (min)	10.3 (2.01)	9.5 (1.69)	1.83	0.07	0.84
Motor Coordination					
KTK Moving Sideways (n/2*20s) **	73.9 (8.96)	63.8 (9.46)	4.12**	0.00	0.92
KTK Jumping Sideways (n/2*15s) *	112.7 (10.18)	105.8 (12.88)	2.14*	0.04	0.07
KTK Walking Backwards (n) **	65.1 (6.93)	55.8 (12.69)	3.00**	0.00	0.01

**indicates a significant difference between groups (p<0.01),

*indicates a trend towards significant (p<0.05).

**Table 4:** Means (SD) and statistics of Z-scores on anthropometric, physical performance and motor coordination tests of elite and non-elite taekwondo athletes.

Measurement	Z-Score (SD)		MANOVA		Partial Eta Squared	Covariate (APHV)		MANCOVA (APHV)	
	Elite	Non Elite	F	p		F	P	F	p
**Anthropometric**			2.872*	**0.027**	**0.11**	6.913**	**0.000**	2.789*	**0.031**
Height	0.21 (0.79)	-0.05 (0.97)	1.075	0.302	0.02	23.061**	0.000	0.607	0.438
Weight	-0.08 (0.89)	0.02 (0.96)	0.166	0.684	0.00	24.007**	0.000	0.685	0.410
Fat Percentage	-0.55 (0.98)	0.12 (0.89)	7.928**	0.006	0.11	4.798*	0.031	9.181**	0.003
BMI	-0.37 (0.85)	0.08 (0.95)	3.440	0.067	0.05	6.437*	0.013	4.389*	0.039
**Physical Performance**			3.331**	**0.006**	**0.22**	**1.178**	**0.198**	3.411**	**0.005**
Sit & Reach	0,26 (0.78)	-0.05 (0.97)	1.694	0.197	0.02	0.532	0.468	1.536	0.219
Sprint 5m	-0.32 (0.87)	0.07 (0.95)	2.889	0.093	0.04	0.762	0.386	3.100	0.082
Sprint 30m	-0.48 (0.91)	0.11 (0.92)	4.963*	0.029	0.06	0.819	0.368	4.628*	0.035
Counter Movement Jump	0.79 (0.73)	-0.18 (0.89)	18.340**	0.000	0.19	0.044	0.835	18.144**	0.000
Squat Jump	0.42 (0.92)	-0.11 (0.92)	4.318*	0.041	0.05	0.578	0.450	4.508*	0.037
Endurance Shuttle Run	0.39 (0.99)	-0.09 (0.91)	2.755	0.101	0.04	1.508	0.223	3.071	0.084
**Motor Coordination**			7.207**	**0.000**	**0.19**	**0.754**	**0.523**	7.343**	**0.000**
KTK Moving Sideways	0.79 (0.74)	-0.18 (0.89)	18.790**	0.000	0.18	0.834	0.363	19.229**	0.000
KTK Jumping Sideways	0.34 (0.78)	-0.08 (0.96)	2.957	0.089	0.03	1.865	0.175	3.314	0.072
KTK Walking Backwards	0.54 (0.66)	-0.12 (0.96)	7.951**	0.006	0.08	1.011	0.317	8.321**	0.005

** indicates a significant difference between groups (p<0.01),

*indicates a trend towards significant (p<0.05).

### Physical performance

Descriptive data (Table 3) show that elite taekwondo athletes score better than non-elite group in all of the physical performance tests. There are significant differences in t-test results for sprint 30m, counter movement jump and squat jump test (Table 3). The higher physical performance scores in Table 4 are reflected in significant overall effect in physical performance (p<0.006). Using the z-score data (Table 4), MANOVA analysis on z-scores show significant differences in physical performance more specifically in the sprint 30 m, counter movement jump and squat jump test. AUCs for these tests were 0.68, 0.82, and 0.70, respectively.

### Motor coordination

Raw scores on motor coordination means appear to be higher in the elite group compared to the non-elite group (Table 3). However, the three general motor coordination tests show significant differences in the t-test results (Table 3). This observation was confirmed by the MANOVA analysis (Table 4) on the z-scores showing significantly better scores for elite athletes in two out of three general motor coordination tests. AUC was 0.80 for moving sideways and 0.71 for the balance beam.

In Table 5, the z-scores obtained from the 18 elite taekwondo athletes have been reconverted into raw performance scores on each test, for male and female athletes between 12 and 17 years of age. For example, a z-score of 0.21 for length which is indicative for elite taekwondo athletes in general, equals 158.38 cm in 12 years old male taekwondo athletes, a figure that rises up to 180.88 cm at the age of 17.

**Table 5:** Converted from z score to raw score (New score).

Age	TKD z-score	12		13		14		15		16		17	
		Male	Female	Male	Female	Male	Female	Male	Female	Male	Female	Male	Female
**Anthropometry**													
Height (cm)	0.21	158.38	158.40	161.84	163.19	173.53	168.24	179.82	167.59	180.77	170.97	180.88	170.86
Weight (kg)	-0.08	42.63	42.84	46.18	47.33	56.01	54.10	63.16	56.76	66.25	58.11	69.68	60.16
Fat Percentage (%)	-0.55	10.74	15.73	9.75	16.27	9.13	18.62	9.11	20.29	9.21	20.06	9.36	20.78
BMI (kg/m^2^)	-0.37	17.03	16.83	17.42	17.70	18.55	18.96	19.50	19.95	20.23	19.97	21.08	20.52
**Physical Performance**													
Sit & Reach (cm)	0.26	25.13	28.23	23.75	31.21	25.82	32.93	28.04	34.21	27.00	33.65	31.27	33.34
Sprint 5m (s)	-0.32	1.21	1.22	1.18	1.19	1.12	1.19	1.09	1.17	1.08	1.14	1.07	1.19
Sprint 30m (s)	-0.48	4.93	5.03	4.84	4.83	4.60	4.82	4.41	4.76	4.34	4.66	4.27	4.77
Counter Movement Jump (cm)	0.79	30.94	28.73	31.11	31.97	35.87	30.68	43.14	32.16	40.43	32.83	42.98	32.93
Endurance Shuttle Run (min)	0.39	10.22	9.42	10.59	9.74	11.47	10.47	12.22	10.12	12.28	9.92	12.87	10.35
**Motor Coordination**													
KTK Moving Sideways (n/2*20s)	0.79	63.83	64.39	65.21	69.06	68.18	70.49	72.36	71.10	74.19	72.28	75.47	71.59
KTK Jumping Sideways (n/2*15s)	0.34	91.92	89.84	93.01	92.34	96.40	97.77	100.23	98.23	101.64	96.61	104.73	98.63
KTK Walking Backwards (n)	0.54	64.82	64.69	70.97	66.60	61.78	65.05	64.83	66.98	64.68	67.88	67.87	67.04

## Discussion

This cross-sectional study was designed to investigate differences in anthropometry, physical performance level, and motor coordination between elite and non-elite taekwondo athletes. Apart from providing reference values in taekwondo athletes, the main finding of this study is that the medal winners in international competitions can be distinguished from sub-elite peers based upon morphological, physiological, and motor coordination characteristics.

Elite taekwondo athletes showed a lower fat percentage compared to their non-elite counterparts. Previous studies also showed significant differences in anthropometric characteristics between combat sport athletes in judo [5,6], karate [6] and taekwondo [6]. Especially in combat sports with weight categories, fat percentage is one of the most important variables. There are expectations that height will be an important factor or an advantage for elite taekwondo athletes as compared to non-elite taekwondo athletes [7,10]; if one has a longer arm or a longer leg they may be able to reach their opponent or contact point much easier and this can be viewed as an advantage for the athlete. However, based on this study, no significant differences were found between those groups in standing height or body stature. Based on previous studies, it shows that taekwondo is a sport with combination of various parameters of physical characteristics [7,15,19,20].

The physical performance tests revealed better scores in speed (5m sprint) and power (CMJ and squat jump) between elite and no-elite taekwondo athletes. Speed and power have indeed been shown to be relevant determinants of taekwondo performance [19]. Especially lower limb power generation is critical for the execution of powerful kicks [12,13].

While the above results generally corroborate literature findings on the topic, the added value of this study is the observation that medal-winning athletes have better general motor coordination. In contrast to sport-specific motor skill tests (like the execution of a specific kick), the KTK test used in the current study is only affected by sport-specific training to a limited extent, which makes it easier to discriminate innate giftedness from tests scores that are the result of training. This finding might lead to the conclusion that general motor coordination is an important factor in determining who makes it into an elite level in taekwondo and who does not. Indeed, general motor coordination has been proven to be a valuable indicator of an athlete’s potential for progression and as such, an important talent characteristic in skill-based sports such as artistic gymnastics [21] and combat sports [5,6]. For example, elite judo athletes showed better motor coordination and balance as compared to their non-elite colleagues [35]. The underlying assumption is that general motor coordination is a foundation on which sport-specific skills are built, and that an athlete with better coordination will learn new techniques faster and easier [21].

The present study identified that young taekwondo athletes can be differentiated by their fat percentage, sprint 30 m, counter movement jump, squat jump, moving sideways and walking backwards ability. Combination of the variables, especially in physical performance can help coaches and strength conditioning professionals to better understand the performance of their athletes and discover the best approach to improve their training organization. More attention should be given to sprint and explosive power training given the significant differences between elite and non-elite in this study. Nevertheless, this variable can also be used as an early form of detection for young talented athletes in martial arts sports especially in that of taekwondo for the future.

Identifying actual performance is possible with physical performance characteristics such as jumping, running or any physical fitness tests that are already used by researchers in many studies [12,15,20]. Actual performance information is helpful for the coaches and very useful information in developing training program for the athletes. Motor coordination measurements are much more important when identifying the future performance potential, especially to identify and discover the potential in young athletes [6,8,22].

The reconversion from z-scores to actual scores by means of a large database of well-trained individuals has, to our knowledge, not been presented before. It might be an innovative way to draw conclusions on attributes of young potentials, based upon a small sample of elites. Such information is very useful for coaches that are involved in the talent selection and identification process. This approach does however have limitations. Assuming that a given average z-score will hold for young athletes of different ages does not reckon with the observation that talent characteristics are dynamic between 12 and 17 years of age. The relative importance of a given characteristic might change during the talent development phases, and also be affected by biological maturation. Researchers have claimed that biological maturation is an important factor in talent programs, especially for young athletes in sport and it show a significant relationship throughout the development process of athletes [36,37,38]. However, in this study biological maturation did not systematically affect expertise-related differences. A second limitation is that the current reference values in young taekwondo athletes are based upon cross-sectional data. Future longitudinal research should be performed to support our findings. Finally, the fact that sex and age were not distributed equally for each age group was another limitation of this study. Such limitations are however inevitably in research on characteristics of young elite athletes, who are low in numbers by definition.

In spite of these limitations, z-scores provide coaches with raw data that are at least indicative for young talented athletes, in the absence of data from large samples of internationally successful athletes.

## Conclusion

This study has managed to distinguish between elite and non-elite among taekwondo athletes in terms of their anthropometric measurements, physical performance tests and motor coordination tests. Especially factors like explosivity and general motor coordination seem to be important discriminants between athletes who reach the international top and those that do not. Taekwondo coaches and federations can use this information in their talent identification and development programs, however keeping in mind that anthropometry, physical performance, and coordination are only partial expressions of talent. Such information can be an added value to the expert opinion of the coach because they seem to know and understand their athletes better compared to others. Competition results and psychological factors are valuable to evaluate athlete progression and effectiveness of the training or development program.

## Supporting information

S1 Table Demographic information of the elite and non-elite taekwondo athletes. (DOC)

S2 Table Data for the counter movement jump for boys in The Elite Sport Schools [23]. (DOC)

S3 Table Mean and standard deviations (Sd) from the descriptive analysis, T-test results and Levene’s test for elite and non-elite taekwondo athletes. (DOC)

S4 Table Means (SD) and statistics of Z-scores on anthropometric, physical performance and motor coordination tests of elite and non-elite taekwondo athletes. (DOC)

S5 Table Converted from z score to raw score (New score). (DOC)

S1 File Raw data. (PDF)
