# Supplementary material for: Identification of elite performance characteristics in a small sample of taekwondo athletes
Source: PLoS One. 2019 May 31;14(5):e0217358. doi: 10.1371/journal.pone.0217358 (PMC6544235; doi:10.1371/journal.pone.0217358)
Supplement: S1 Table — (DOC) [file pone.0217358.s001.doc]

**Table 1: Demographic Information of The Elite and Non-Elite Taekwondo Athletes.**

| **Age Group (years)** | **Elite** | | **Non Elite** | | **Total** |
| --- | --- | --- | --- | --- | --- |
|  | **Male** | **Female** | **Male** | **Female** |  |
| 12 | 1 | 1 | 8 | 2 | 12 |
| 13 | 1 | 1 | 7 | 6 | 15 |
| 14 | 3 | 2 | 10 | 10 | 25 |
| 15 | 2 | 2 | 7 | 12 | 23 |
| 16 | 1 | 2 | 10 | 3 | 16 |
| 17 | 1 | 1 | 1 | 4 | 7 |
| Total | 9 | 9 | 43 | 37 | 98 |
