# Supplementary material for: Identification of elite performance characteristics in a small sample of taekwondo athletes
Source: PLoS One. 2019 May 31;14(5):e0217358. doi: 10.1371/journal.pone.0217358 (PMC6544235; doi:10.1371/journal.pone.0217358)
Supplement: S2 Table — (DOC) [file pone.0217358.s002.doc]

**Table 2:Data for The Counter Movement Jump for Boys in The Elite Sport Schools [23]**

| **Age (years)** | **N** | **Minimum (cm)** | **Maximum (cm)** | **Mean (cm)** | **SD (cm)** |
| --- | --- | --- | --- | --- | --- |
|  |  |  |  |  |  |
| 12 | 48 | 18.80 | 38.85 | 27.53 | 4.30 |
| 13 | 60 | 17.60 | 38.00 | 27.78 | 4.21 |
| 14 | 74 | 19.42 | 51.63 | 31.68 | 5.30 |
| 15 | 74 | 23.2 | 54.70 | 34.84 | 5.44 |
| 16 | 82 | 24.38 | 51.36 | 35.79 | 5.87 |
| 17 | 87 | 25.8 | 56.51 | 37.81 | 6.55 |

The data based on the CMJ score from the Flemish Top Sport School athlete in gymnastic, skating, soccer, athletics, badminton, basketball, handball, judo, fencing, taekwondo, table tennis, triathlon, tennis, golf, volleyball, swimming, cycling, ski.
