# Supplementary material for: Identification of elite performance characteristics in a small sample of taekwondo athletes
Source: PLoS One. 2019 May 31;14(5):e0217358. doi: 10.1371/journal.pone.0217358 (PMC6544235; doi:10.1371/journal.pone.0217358)
Supplement: S3 Table — (DOC) [file pone.0217358.s003.doc]

**Table 3: Mean and Standard Deviations (Sd) from The Descriptive Analysis, T-Test Results and Levene’s Test for Elite and Non-Elite Taekwondo Athletes.**

| **Measurement** | **Mean (SD)** | | **t-Test result** | | **Levene’s Test** |
| --- | --- | --- | --- | --- | --- |
|  | **Elite** | **Non-Elite** | **t** | **p** | **p** |
|  |  |  |  |  |  |
| Anthropometry |  |  |  |  |  |
| Height (cm) | 166.7 (8.28) | 163.4 (10.49) | 1.28 | 0.20 | 0.18 |
| Weight (kg) | 51.3 (9.00) | 51.9 (10.64) | -0.19 | 0.85 | 0.42 |
| Fat Percentage (%) * | 11.9 (3.65) | 15.12 (6.55) | -2.01* | 0.05 | 0.00 |
| BMI (kg/m²) | 18.3 (1.78) | 19.3 (2.38) | -1.59 | 0.12 | 1.22 |
|  |  |  |  |  |  |
| Physical Performance |  |  |  |  |  |
| Sit & Reach (cm) | 32.5 (6.29) | 30.1 (7.87) | 1.22 | 0.23 | 4.22 |
| Sprint 5m (s) | 1.16 (0.08) | 1.19 (0.09) | -1.59 | 0.12 | 0.72 |
| Sprint 30m (s) * | 4.72 (0.33) | 4.92 (0.37) | -2.11* | 0.04 | 0.66 |
| Counter Movement Jump (cm) ** | 33.4 (4.95) | 28.4 (5.62) | 3.50** | 0.00 | 0.84 |
| Squat Jump (cm) * | 30.1 (6.45) | 26.4 (5.19) | 2.49* | 0.02 | 0.47 |
| Endurance Shuttle Run (min) | 10.3 (2.01) | 9.5 (1.69) | 1.83 | 0.07 | 0.84 |
|  |  |  |  |  |  |
| Motor Coordination |  |  |  |  |  |
| KTK Moving Sideways (n/2*20s) ** | 73.9 (8.96) | 63.8 (9.46) | 4.12** | 0.00 | 0.92 |
| KTK Jumping Sideways (n/2*15s) * | 112.7 (10.18) | 105.8 (12.88) | 2.14* | 0.04 | 0.07 |
| KTK Walking Backwards (n) ** | 65.1 (6.93) | 55.8 (12.69) | 3.00** | 0.00 | 0.01 |

**indicates a significant difference between groups (p<0.01), *indicates a trend towards significant (p<0.05).
