# Supplementary material for: Identification of elite performance characteristics in a small sample of taekwondo athletes
Source: PLoS One. 2019 May 31;14(5):e0217358. doi: 10.1371/journal.pone.0217358 (PMC6544235; doi:10.1371/journal.pone.0217358)
Supplement: S4 Table — (DOC) [file pone.0217358.s004.doc]

**Table 4: Means (SD) and Statistics of Z-Scores on Anthropometric, Physical Performance and Motor Coordination Tests of Elite and Non-Elite Taekwondo Athletes.**

| **Measurement** | **Z-Score (SD)** | | **MANOVA** | | **Partial Eta Squared** | **Covariate**  **(APHV)** | | **MANCOVA**  **(APHV)** | |
| --- | --- | --- | --- | --- | --- | --- | --- | --- | --- |
|  | **Elite** | **Non Elite** | **F** | **p** |  | **F** | **P** | **F** | **p** |
|  |  |  |  |  |  |  |  |  |  |
| **Anthropometric** |  |  | **2.872*** | **0.027** | **0.11** | **6.913**** | **0.000** | **2.789*** | **0.031** |
| Height | 0.21 (0.79) | -0.05 (0.97) | 1.075 | 0.302 | 0.02 | 23.061** | 0.000 | 0.607 | 0.438 |
| Weight | -0.08 (0.89) | 0.02 (0.96) | 0.166 | 0.684 | 0.00 | 24.007** | 0.000 | 0.685 | 0.410 |
| Fat Percentage | -0.55 (0.98) | 0.12 (0.89) | 7.928** | 0.006 | 0.11 | 4.798* | 0.031 | 9.181** | 0.003 |
| BMI | -0.37 (0.85) | 0.08 (0.95) | 3.440 | 0.067 | 0.05 | 6.437* | 0.013 | 4.389* | 0.039 |
|  |  |  |  |  |  |  |  |  |  |
| **Physical Performance** |  |  | **3.331**** | **0.006** | **0.22** | **1.178** | **0.198** | **3.411**** | **0.005** |
| Sit & Reach | 0,26 (0.78) | -0.05 (0.97) | 1.694 | 0.197 | 0.02 | 0.532 | 0.468 | 1.536 | 0.219 |
| Sprint 5m | -0.32 (0.87) | 0.07 (0.95) | 2.889 | 0.093 | 0.04 | 0.762 | 0.386 | 3.100 | 0.082 |
| Sprint 30m | -0.48 (0.91) | 0.11 (0.92) | 4.963* | 0.029 | 0.06 | 0.819 | 0.368 | 4.628* | 0.035 |
| Counter Movement Jump | 0.79 (0.73) | -0.18 (0.89) | 18.340** | 0.000 | 0.19 | 0.044 | 0.835 | 18.144** | 0.000 |
| Squat Jump | 0.42 (0.92) | -0.11 (0.92) | 4.318* | 0.041 | 0.05 | 0.578 | 0.450 | 4.508* | 0.037 |
| Endurance Shuttle Run | 0.39 (0.99) | -0.09 (0.91) | 2.755 | 0.101 | 0.04 | 1.508 | 0.223 | 3.071 | 0.084 |
|  |  |  |  |  |  |  |  |  |  |
| **Motor Coordination** |  |  | **7.207**** | **0.000** | **0.19** | **0.754** | **0.523** | **7.343**** | **0.000** |
| KTK Moving Sideways | 0.79 (0.74) | -0.18 (0.89) | 18.790** | 0.000 | 0.18 | 0.834 | 0.363 | 19.229** | 0.000 |
| KTK Jumping Sideways | 0.34 (0.78) | -0.08 (0.96) | 2.957 | 0.089 | 0.03 | 1.865 | 0.175 | 3.314 | 0.072 |
| KTK Walking Backwards | 0.54 (0.66) | -0.12 (0.96) | 7.951** | 0.006 | 0.08 | 1.011 | 0.317 | 8.321** | 0.005 |

**indicates a significant difference between groups (p<0.01), *indicates a trend towards significant (p<0.05).
