# Supplementary material for: Identification of elite performance characteristics in a small sample of taekwondo athletes
Source: PLoS One. 2019 May 31;14(5):e0217358. doi: 10.1371/journal.pone.0217358 (PMC6544235; doi:10.1371/journal.pone.0217358)
Supplement: S5 Table — (DOC) [file pone.0217358.s005.doc]

Table 5: Converted from z score to raw score (New Score)

| **Age** |  | **12** | | **13** | | **14** | | **15** | | **16** | | **17** | |
| --- | --- | --- | --- | --- | --- | --- | --- | --- | --- | --- | --- | --- | --- |
|  | TKD z-score | Male | Female | Male | Female | Male | Female | Male | Female | Male | Female | Male | Female |
| **Anthropometry** |  |  |  |  |  |  |  |  |  |  |  |  |  |
| Height (cm) | 0.21 | 158.38 | 158.40 | 161.84 | 163.19 | 173.53 | 168.24 | 179.82 | 167.59 | 180.77 | 170.97 | 180.88 | 170.86 |
| Weight (kg) | -0.08 | 42.63 | 42.84 | 46.18 | 47.33 | 56.01 | 54.10 | 63.16 | 56.76 | 66.25 | 58.11 | 69.68 | 60.16 |
| Fat Percentage (%) | -0.55 | 10.74 | 15.73 | 9.75 | 16.27 | 9.13 | 18.62 | 9.11 | 20.29 | 9.21 | 20.06 | 9.36 | 20.78 |
| BMI (kg/m²) | -0.37 | 17.03 | 16.83 | 17.42 | 17.70 | 18.55 | 18.96 | 19.50 | 19.95 | 20.23 | 19.97 | 21.08 | 20.52 |
|  |  |  |  |  |  |  |  |  |  |  |  |  |  |
| **Physical Performance** |  |  |  |  |  |  |  |  |  |  |  |  |  |
| Sit & Reach (cm) | 0.26 | 25.13 | 28.23 | 23.75 | 31.21 | 25.82 | 32.93 | 28.04 | 34.21 | 27.00 | 33.65 | 31.27 | 33.34 |
| Sprint 5m (s) | -0.32 | 1.21 | 1.22 | 1.18 | 1.19 | 1.12 | 1.19 | 1.09 | 1.17 | 1.08 | 1.14 | 1.07 | 1.19 |
| Sprint 30m (s) | -0.48 | 4.93 | 5.03 | 4.84 | 4.83 | 4.60 | 4.82 | 4.41 | 4.76 | 4.34 | 4.66 | 4.27 | 4.77 |
| Counter Movement Jump (cm) | 0.79 | 30.94 | 28.73 | 31.11 | 31.97 | 35.87 | 30.68 | 43.14 | 32.16 | 40.43 | 32.83 | 42.98 | 32.93 |
| Endurance Shuttle Run (min) | 0.39 | 10.22 | 9.42 | 10.59 | 9.74 | 11.47 | 10.47 | 12.22 | 10.12 | 12.28 | 9.92 | 12.87 | 10.35 |
|  |  |  |  |  |  |  |  |  |  |  |  |  |  |
| **Motor Coordination** |  |  |  |  |  |  |  |  |  |  |  |  |  |
| KTK Moving Sideways (n/2*20s) | 0.79 | 63.83 | 64.39 | 65.21 | 69.06 | 68.18 | 70.49 | 72.36 | 71.10 | 74.19 | 72.28 | 75.47 | 71.59 |
| KTK Jumping Sideways (n/2*15s) | 0.34 | 91.92 | 89.84 | 93.01 | 92.34 | 96.40 | 97.77 | 100.23 | 98.23 | 101.64 | 96.61 | 104.73 | 98.63 |
| KTK Walking Backwards (n) | 0.54 | 64.82 | 64.69 | 70.97 | 66.60 | 61.78 | 65.05 | 64.83 | 66.98 | 64.68 | 67.88 | 67.87 | 67.04 |
