# Supplementary material for: Identification of elite performance characteristics in a small sample of taekwondo athletes
Source: PLoS One. 2019 May 31;14(5):e0217358. doi: 10.1371/journal.pone.0217358 (PMC6544235; doi:10.1371/journal.pone.0217358)
Supplement: S1 File — (PDF) [file pone.0217358.s006.pdf]

| ID | Group | Gender | Sex | Age Group | Height | Sitting Height | Fat Percentage | BMI | Sit & reach | Sprint 5m | Sprint 30m | Counter Movement Jump | Squat jump | Beep test | Moving Sideways | Jumping Sideways | Balance beam | APHV |
|---|---|---|---|---|---|---|---|---|---|---|---|---|---|---|---|---|---|---|
| 1 | 0 | 0 | Female | 12 | 151.3 | 35.6 | 12.2 | 15.55 | | 1.171 | 5.075 | 30.4 | | 11.0 | 67 | 103 | 71 | 12.44 |
| 2 | 0 | 1 | Male | 12 | 149.7 | 39.6 | 12.3 | 17.67 | 26.0 | 1.217 | 5.214 | 24.1 | 22.6 | 8.5 | 67 | 103 | 66 | 14.25 |
| 3 | 0 | 0 | Female | 13 | 154.9 | 37.1 | 9.0 | 15.46 | 34.0 | 1.195 | 5.040 | 30.2 | 28.0 | 10.5 | 69 | 104 | 71 | 12.45 |
| 4 | 0 | 1 | Male | 13 | 159.6 | 41.4 | 11.4 | 16.25 | 35.5 | 1.239 | 5.042 | 27.9 | 27.2 | 11.0 | 65 | 108 | 72 | 14.19 |
| 5 | 0 | 0 | Female | 14 | 173.8 | 53.9 | 17.4 | 17.84 | 37.0 | 1.242 | 4.585 | 33.1 | 26.6 | 10.0 | 78 | 114 | 67 | 12.09 |
| 6 | 0 | 0 | Female | 14 | 165.8 | 46.9 | 10.7 | 17.06 | 29.5 | 1.108 | 4.699 | 38.2 | 13.0 | 4.5 | 61 | 101 | 59 | 12.33 |
| 7 | 0 | 1 | Male | 14 | 174.5 | 63.5 | 13.5 | 20.85 | 24.5 | 1.183 | 4.911 | 28.2 | 27.3 | 9.0 | 66 | 110 | 44 | 13.52 |
| 8 | 0 | 1 | Male | 14 | 170.1 | 57.4 | 16.8 | 19.84 | 28.5 | 1.139 | 4.637 | 31.6 | 31.6 | 13.0 | 62 | 99 | 62 | 13.75 |
| 9 | 0 | 1 | Male | 14 | 167.8 | 55.4 | 6.2 | 19.68 | 31.5 | 1.047 | 4.180 | 40.0 | 34.0 | 9.5 | 72 | 112 | 58 | 13.57 |
| 10 | 0 | 0 | Female | 15 | 166.2 | 48.5 | 12.0 | 17.56 | 40.0 | 1.152 | 4.880 | 30.2 | 28.6 | 12.5 | 79 | 110 | 72 | 12.55 |
| 11 | 0 | 0 | Female | 15 | 161.5 | 46.1 | 14.8 | 17.67 | 41.0 | 1.109 | 4.688 | 28.7 | 27.2 | 9.5 | 71 | 111 | 68 | 12.89 |
| 12 | 0 | 1 | Male | 15 | 179.1 | 63.7 | 12.7 | 19.86 | 23.0 | 1.112 | 4.681 | 34.8 | 31.9 | 11.0 | 74 | 141 | 66 | 13.65 |
| 13 | 0 | 1 | Male | 15 | 170.0 | 55.9 | 5.3 | 19.34 | 37.0 | 1.089 | 4.263 | 39.4 | 36.9 | 10.5 | 76 | 119 | 66 | 13.85 |
| 14 | 0 | 0 | Female | 16 | 170.5 | 52.1 | 13.0 | 17.92 | 40.0 | 1.344 | 5.218 | 31.1 | 30.3 | 11.0 | 82 | 118 | 67 | 12.68 |
| 15 | 0 | 0 | Female | 16 | 172.8 | 55.6 | 15.0 | 18.62 | 34.5 | 1.140 | 4.567 | 38.0 | 33.3 | 10.0 | 91 | 113 | 68 | 12.63 |
| 16 | 0 | 1 | Male | 16 | 168.1 | 48.4 | 4.9 | 17.13 | 20.0 | 1.049 | 4.179 | 40.4 | 37.4 | 12.0 | 86 | 121 | 70 | 15.13 |
| 17 | 0 | 0 | Female | 17 | 174.5 | 58.5 | 15.6 | 19.21 | 35.0 | 1.213 | 4.703 | 35.6 | 34.8 | 9.0 | 89 | 126 | 68 | 14.35 |
| 18 | 0 | 1 | Male | 17 | 171.2 | 64.6 | 12.3 | 22.04 | 35.5 | 1.045 | 4.353 | 39.1 | 41.1 | 13.5 | 76 | 117 | 58 | 14.51 |
| 19 | 1 | 0 | Female | 12 | 163.8 | 49.7 | 20.2 | 18.52 | 35.5 | 1.128 | 4.805 | 26.6 | 25.3 | 9.5 | 59 | 97 | 51 | 14.40 |
| 20 | 1 | 0 | Female | 12 | 169.7 | 48.9 | 15.0 | 16.98 | 26.0 | 1.313 | 5.572 | 23.8 | 21.3 | 8.0 | 50 | 89 | 31 | 11.50 |
| 21 | 1 | 1 | Male | 12 | 153.9 | 39.3 | 8.9 | 16.59 | 19.0 | 1.272 | 5.668 | 25.8 | 22.6 | 7.0 | 46 | 80 | 35 | 13.86 |
| 22 | 1 | 1 | Male | 12 | 152.5 | 34.4 | 6.1 | 14.79 | 16.5 | 1.263 | 5.181 | 31.6 | 29.4 | 8.0 | 49 | 91 | 49 | 14.01 |
| 23 | 1 | 1 | Male | 12 | 148.3 | 46.3 | 21.5 | 21.05 | 31.0 | 1.316 | 5.443 | 23.8 | 21.9 | 5.0 | 54 | 87 | 37 | 13.67 |
| 24 | 1 | 1 | Male | 12 | 149.6 | 36.2 | 11.3 | 16.18 | 29.0 | 1.096 | 4.945 | 25.2 | 24.5 | 11.0 | 61 | 112 | 70 | 14.37 |

| | | | | | | | | | | | | | | | | | | |
|---|---|---|---|---|---|---|---|---|---|---|---|---|---|---|---|---|---|---|
| 25 | 1 | 1 | Male | 12 | 144.0 | 33.2 | 9.8 | 16.01 | 38.0 | 1.274 | 5.255 | 30.2 | 28.7 | 6.5 | 64 | 95 | 72 | 14.27 |
| 26 | 1 | 1 | Male | 12 | 149.4 | 40.9 | 18.7 | 18.32 | 30.0 | 1.276 | 5.517 | 21.8 | 21.3 | 8.5 | 60 | 89 | 66 | 13.66 |
| 27 | 1 | 1 | Male | 12 | 152.4 | 38.4 | 13.9 | 16.53 | 6.0 | 1.274 | 5.865 | 20.6 | 20.6 | 8.5 | 50 | 88 | 52 | 13.86 |
| 28 | 1 | 1 | Male | 12 | 158.2 | 64.8 | 29.1 | 25.89 | 15.0 | 1.349 | 5.675 | 18.8 | 14.9 | 7.0 | 54 | 83 | 34 | 13.16 |
| 29 | 1 | 0 | Female | 13 | 160.2 | 46.0 | 16.2 | 17.92 | 23.0 | 1.231 | 4.954 | 25.5 | 24.6 | 9.5 | 61 | 102 | 55 | 12.33 |
| 30 | 1 | 0 | Female | 13 | 157.5 | 42.2 | 11.8 | 17.01 | 31.0 | 1.127 | 4.668 | 29.4 | 25.2 | 10.0 | 53 | 102 | 54 | 12.49 |
| 31 | 1 | 0 | Female | 13 | 167.7 | 56.2 | 22.0 | 19.98 | 41.0 | 1.338 | 4.904 | 25.9 | 24.6 | 8.5 | 65 | 116 | 54 | 11.53 |
| 32 | 1 | 0 | Female | 13 | 154.7 | 44.7 | 19.3 | 18.68 | 35.0 | 1.184 | 4.888 | 31.9 | 28.2 | 11.0 | 61 | 115 | 51 | 12.67 |
| 33 | 1 | 0 | Female | 13 | 154.5 | 39.2 | 15.8 | 16.42 | 24.0 | 1.195 | 4.909 | 23.8 | 25.2 | 8.0 | 56 | 80 | 53 | 12.32 |
| 34 | 1 | 0 | Female | 13 | 142.1 | 32.1 | 13.3 | 15.90 | 29.0 | 1.180 | 5.135 | 24.5 | 23.8 | 10.0 | 51 | 101 | 41 | 13.46 |
| 35 | 1 | 1 | Male | 13 | 166.7 | 54.6 | 10.4 | 19.65 | 17.0 | 1.264 | 5.489 | 23.8 | 24.5 | 6.0 | 50 | 96 | 51 | 13.46 |
| 36 | 1 | 1 | Male | 13 | 151.3 | 40.9 | 11.2 | 17.87 | | 1.230 | 5.323 | 29.0 | | 12.5 | 59 | 112 | 50 | 14.57 |
| 37 | 1 | 1 | Male | 13 | 151.3 | 40.6 | 10.9 | 17.74 | | 1.165 | 4.794 | 35.7 | | 12.0 | 56 | 112 | 50 | 14.34 |
| 38 | 1 | 1 | Male | 13 | 143.4 | 44.5 | 28.5 | 21.64 | 29.0 | 1.197 | 5.191 | 24.5 | 23.8 | 7.5 | 56 | 99 | 41 | 14.66 |
| 39 | 1 | 1 | Male | 13 | 157.0 | 50.5 | 15.2 | 20.49 | 24.0 | 1.283 | 5.320 | 25.2 | 20.0 | 9.5 | 58 | 93 | 56 | 13.74 |
| 40 | 1 | 1 | Male | 13 | 163.8 | 45.4 | 12.9 | 16.92 | 22.0 | 1.244 | 5.046 | 25.2 | 27.2 | 10.5 | 54 | 103 | 54 | 13.62 |
| 41 | 1 | 1 | Male | 13 | 147.9 | 34.7 | 7.4 | 15.86 | 29.0 | 1.154 | 5.013 | 25.3 | 25.2 | 7.5 | 64 | 113 | 66 | 14.51 |
| 42 | 1 | 0 | Female | 14 | 168.2 | 53.9 | 23.4 | 19.05 | 32.0 | 1.133 | 4.750 | 23.2 | 24.5 | 10.0 | 57 | 93 | 65 | 12.28 |
| 43 | 1 | 0 | Female | 14 | 151.2 | 47.0 | 25.4 | 20.56 | 39.5 | 1.233 | 5.086 | 20.6 | | 9.0 | 74 | 118 | 49 | 13.11 |
| 44 | 1 | 0 | Female | 14 | 161.5 | 44.0 | 14.3 | 16.87 | 30.0 | 1.193 | 4.997 | 27.9 | | | 70 | 114 | 51 | 12.61 |
| 45 | 1 | 0 | Female | 14 | 166.0 | 50.8 | 20.6 | 18.44 | 28.0 | 1.257 | 5.339 | 21.3 | 21.8 | 6.0 | 50 | 71 | 35 | 12.34 |
| 46 | 1 | 0 | Female | 14 | 173.7 | 62.3 | 23.5 | 20.65 | 29.0 | 1.187 | 4.920 | 23.8 | 22.6 | 8.0 | 62 | 87 | 59 | 11.85 |
| 47 | 1 | 0 | Female | 14 | 159.1 | 47.7 | 20.2 | 18.84 | 33.0 | 1.215 | 4.862 | 28.0 | 28.0 | 8.5 | 63 | 105 | 70 | 14.83 |
| 48 | 1 | 0 | Female | 14 | 146.3 | 33.5 | 11.9 | 15.65 | 26.0 | 1.160 | 5.192 | 22.6 | 25.9 | 8.5 | 58 | 107 | 66 | 13.41 |
| 49 | 1 | 0 | Female | 14 | 167.6 | 51.2 | 20.7 | 18.23 | 31.5 | 1.259 | 5.356 | 24.5 | 22.6 | 9.0 | 64 | 97 | 56 | 12.05 |

| | | | | | | | | | | | | | | | | | | |
|---|---|---|---|---|---|---|---|---|---|---|---|---|---|---|---|---|---|---|
| 50 | 1 | 0 | Female | 14 | 158.5 | 63.4 | 31.9 | 25.24 | 38.0 | 1.139 | 4.845 | 23.8 | 25.2 | 8.0 | 66 | 105 | 63 | 12.73 |
| 51 | 1 | 0 | Female | 14 | 148.3 | 37.5 | 14.6 | 17.05 | 27.0 | 1.269 | 5.329 | 25.2 | 23.2 | 9.5 | 62 | 101 | 62 | 13.40 |
| 52 | 1 | 1 | Male | 14 | 178.0 | 49.5 | 3.9 | 15.62 | 12.0 | 1.155 | 4.985 | 22.0 | 20.6 | 7.0 | 56 | 103 | 33 | 13.75 |
| 53 | 1 | 1 | Male | 14 | 163.1 | 54.9 | 17.3 | 20.64 | 36.5 | 1.073 | 4.373 | 38.2 | 39.0 | 9.5 | 63 | 93 | 66 | 13.37 |
| 54 | 1 | 1 | Male | 14 | 161.5 | 38.6 | 5.9 | 14.80 | 24.0 | 1.164 | 5.078 | 28.7 | 30.4 | 12.0 | 59 | 89 | 68 | 14.87 |
| 55 | 1 | 1 | Male | 14 | 163.5 | 49.5 | 9.0 | 18.52 | 29.0 | 1.169 | 4.730 | 39.8 | 38.0 | 12.5 | 60 | 120 | 57 | 11.49 |
| 56 | 1 | 1 | Male | 14 | 144.8 | 38.4 | 6.9 | 18.31 | 21.0 | 1.187 | 4.898 | 35.6 | 32.6 | 9.0 | 66 | 108 | 63 | 15.05 |
| 57 | 1 | 1 | Male | 14 | 164.9 | 54.5 | 9.1 | 20.04 | 21.5 | 1.161 | 4.855 | 25.2 | 23.8 | 11.0 | 61 | 107 | 57 | 13.82 |
| 58 | 1 | 1 | Male | 14 | 166.5 | 50.0 | 9.0 | 18.04 | 32.0 | 1.136 | 4.488 | 36.4 | 32.9 | 13.0 | 75 | 126 | 72 | 14.35 |
| 59 | 1 | 1 | Male | 14 | 165.8 | 52.8 | 13.0 | 19.21 | 35.0 | 1.151 | 4.592 | 31.8 | 24.6 | 9.5 | 72 | 110 | 69 | 12.93 |
| 60 | 1 | 1 | Male | 14 | 175.3 | 57.1 | 13.3 | 18.58 | 20.5 | 1.090 | 4.630 | 23.2 | 21.2 | 11.0 | 64 | 97 | 32 | 13.67 |
| 61 | 1 | 1 | Male | 14 | 162.0 | 44.8 | 4.5 | 17.07 | 24.0 | 1.103 | 4.816 | 33.3 | 29.0 | 11.0 | 69 | 115 | 49 | 14.76 |
| 62 | 1 | 0 | Female | 15 | 165.3 | 59.2 | 25.9 | 21.67 | 28.5 | 1.200 | 5.056 | 23.4 | | 11.0 | 62 | 120 | 65 | 12.50 |
| 63 | 1 | 0 | Female | 15 | 175.3 | 63.4 | 21.4 | 20.63 | 35.0 | 1.290 | 4.926 | 27.3 | 28.7 | 9.5 | 66 | 100 | 61 | 11.78 |
| 64 | 1 | 0 | Female | 15 | 168.5 | 57.3 | 24.5 | 20.18 | 35.0 | 1.183 | 5.029 | 20.8 | 21.2 | 8.0 | 66 | 98 | 60 | 12.61 |
| 65 | 1 | 0 | Female | 15 | 150.4 | 49.5 | 26.3 | 21.88 | 40.5 | 1.414 | 5.341 | 20.9 | 17.9 | 10.0 | 72 | 122 | 62 | 13.41 |
| 66 | 1 | 0 | Female | 15 | 164.7 | 47.4 | 14.1 | 17.47 | 32.5 | 1.145 | 4.760 | 27.9 | | 7.5 | 80 | 121 | 59 | 12.77 |
| 67 | 1 | 0 | Female | 15 | 171.0 | 62.8 | 24.6 | 21.48 | 42.0 | 1.344 | 4.940 | 28.5 | 26.7 | 10.0 | 83 | 120 | 72 | 12.26 |
| 68 | 1 | 0 | Female | 15 | 159.7 | 51.0 | 21.7 | 20.00 | | 1.214 | 5.095 | 24.6 | | 10.0 | 64 | 115 | 41 | 12.91 |
| 69 | 1 | 0 | Female | 15 | 154.4 | 39.1 | 13.8 | 16.40 | 32.0 | 1.098 | 4.958 | 28.7 | | | 63 | 100 | 45 | 13.50 |
| 70 | 1 | 0 | Female | 15 | 157.8 | 43.1 | 14.1 | 17.31 | 31.0 | 1.195 | 5.101 | 27.2 | | 8.5 | 74 | 108 | 68 | 13.54 |
| 71 | 1 | 0 | Female | 15 | 165.1 | 58.8 | 24.4 | 21.57 | 33.0 | 1.202 | 4.784 | 28.0 | 25.2 | 8.5 | 58 | 110 | 68 | 12.57 |
| 72 | 1 | 0 | Female | 15 | 165.7 | 61.2 | 25.1 | 22.29 | 31.0 | 1.325 | 4.881 | 28.1 | 26.3 | 9.5 | 66 | 118 | 55 | 12.82 |
| 73 | 1 | 0 | Female | 15 | 156.5 | 48.3 | 20.2 | 19.72 | 35.0 | 1.124 | 4.412 | 34.0 | 37.2 | 12.0 | 80 | 129 | 72 | 13.28 |
| 74 | 1 | 1 | Male | 15 | 183.0 | 55.1 | 3.1 | 16.45 | 17.0 | 1.164 | 4.768 | 29.4 | 24.3 | 10.0 | 68 | 121 | 46 | 13.61 |

| | | | | | | | | | | | | | | | | | | |
|---|---|---|---|---|---|---|---|---|---|---|---|---|---|---|---|---|---|---|
| 75 | 1 | 1 | Male | 15 | 169.3 | 46.1 | 6.7 | 16.08 | 26.0 | 1.248 | 5.238 | 30.2 | 23.2 | 10.0 | 62 | 94 | 66 | 14.70 |
| 76 | 1 | 1 | Male | 15 | 163.4 | 53.8 | 11.9 | 20.15 | 32.0 | 1.098 | 4.547 | 29.4 | 28.7 | 12.0 | 70 | 120 | 51 | 14.27 |
| 77 | 1 | 1 | Male | 15 | 170.4 | 62.4 | 10.3 | 21.49 | 34.5 | 1.094 | 4.388 | 38.4 | | 11.0 | 67 | 119 | 57 | 13.65 |
| 78 | 1 | 1 | Male | 15 | 172.6 | 61.0 | 10.6 | 20.48 | 33.0 | 1.074 | 4.318 | 28.0 | 38.0 | 10.5 | 66 | 101 | 65 | 13.73 |
| 79 | 1 | 1 | Male | 15 | 172.6 | 56.2 | 8.8 | 18.86 | 38.5 | 0.985 | 4.337 | 44.1 | | | 78 | 105 | 54 | 14.32 |
| 80 | 1 | 1 | Male | 15 | 177.2 | 60.0 | 14.1 | 19.11 | 26.0 | 1.177 | 4.619 | 27.3 | 28.0 | 11.5 | 66 | 106 | 43 | 13.91 |
| 81 | 1 | 0 | Female | 16 | 165.8 | 52.5 | 17.8 | 19.10 | 35.0 | 1.127 | 4.865 | 24.5 | | 8.0 | 77 | 118 | 68 | 13.31 |
| 82 | 1 | 0 | Female | 16 | 170.5 | 60.1 | 15.1 | 20.67 | 42.5 | 1.200 | 4.743 | 27.2 | 25.6 | 9.0 | 92 | 122 | 64 | 12.82 |
| 83 | 1 | 0 | Female | 16 | 158.5 | 49.0 | 16.5 | 19.50 | 28.0 | 1.279 | 5.155 | 23.9 | 21.8 | 9.5 | 84 | 124 | 70 | 13.73 |
| 84 | 1 | 1 | Male | 16 | 183.8 | 77.5 | 13.5 | 22.94 | 17.0 | 1.183 | 4.908 | 23.3 | 19.8 | 8.0 | 49 | 96 | 5 | 13.62 |
| 85 | 1 | 1 | Male | 16 | 182.3 | 70.9 | 10.7 | 21.33 | 32.0 | 1.239 | 4.563 | 36.4 | | 11.0 | 56 | 96 | 54 | 13.36 |
| 86 | 1 | 1 | Male | 16 | 171.0 | 67.7 | 10.9 | 23.15 | 43.0 | | | 34.2 | 33.4 | | 71 | | 72 | 14.42 |
| 87 | 1 | 1 | Male | 16 | 172.4 | 65.3 | 12.8 | 21.97 | 36.0 | 1.156 | 4.335 | 34.8 | 30.9 | 12.0 | 59 | 132 | 72 | 14.12 |
| 88 | 1 | 1 | Male | 16 | 172.7 | 56.2 | 8.5 | 18.84 | 21.0 | 1.094 | 4.311 | 38.9 | 36.4 | 11.0 | 60 | 98 | 56 | 12.69 |
| 89 | 1 | 1 | Male | 16 | 172.2 | 66.7 | 9.8 | 22.49 | 34.0 | 1.092 | 4.582 | 33.3 | 28.7 | 10.0 | 79 | 121 | 72 | 13.76 |
| 90 | 1 | 1 | Male | 16 | 173.8 | 57.5 | 9.3 | 19.04 | 22.0 | 1.041 | 4.531 | 33.3 | 27.3 | 10.0 | 53 | 116 | 38 | 14.11 |
| 91 | 1 | 1 | Male | 16 | 180.5 | 67.6 | 8.2 | 20.75 | 25.0 | 1.119 | 4.314 | 33.7 | 32.0 | 11.0 | 60 | 114 | 50 | 14.27 |
| 92 | 1 | 1 | Male | 16 | 173.7 | 69.1 | 10.6 | 22.90 | 41.0 | 1.033 | 4.294 | 33.3 | 32.5 | 12.0 | 81 | 123 | 46 | 13.97 |
| 93 | 1 | 1 | Male | 16 | 185.3 | 75.5 | 13.9 | 21.99 | 45.0 | 1.029 | 3.989 | 45.5 | 37.5 | 10.0 | 69 | 116 | 67 | 13.79 |
| 94 | 1 | 0 | Female | 17 | 171.6 | 61.6 | 22.9 | 20.92 | 42.0 | 1.375 | 5.056 | 27.8 | 24.6 | 10.0 | 76 | 120 | 71 | 13.11 |
| 95 | 1 | 0 | Female | 17 | 161.2 | 57.9 | 23.3 | 22.28 | 40.5 | 1.226 | 5.217 | 25.2 | | | 60 | 96 | 68 | 13.84 |
| 96 | 1 | 0 | Female | 17 | 160.0 | 47.1 | 10.6 | 18.40 | 35.0 | 1.160 | 4.847 | 30.2 | | 9.0 | 73 | 108 | 53 | 13.93 |
| 97 | 1 | 0 | Female | 17 | 160.2 | 53.2 | 18.2 | 20.73 | 34.5 | 1.170 | 5.186 | 26.8 | 23.8 | 8.0 | 79 | 111 | 52 | 14.09 |
| 98 | 1 | 1 | Male | 17 | 183.9 | 73.7 | 11.0 | 21.79 | 35.5 | 1.125 | 4.450 | 37.2 | | 10.0 | 58 | 101 | 65 | 13.82 |
